# Pancreatic Cancer Mimicking Relapse of Autoimmune Pancreatitis: Case Reports

**DOI:** 10.70352/scrj.cr.25-0076

**Published:** 2025-07-15

**Authors:** Hideaki Kojima, Minoru Kitago, Eisuke Iwasaki, Yohei Masugi, Yuta Abe, Yasushi Hasegawa, Shutaro Hori, Masayuki Tanaka, Yutaka Nakano, Motonori Edanami, Akihisa Ueno, Yuko Kitagawa

**Affiliations:** 1 Department of Surgery, Keio University School of Medicine, Tokyo, Japan; 2 Division of Gastroenterology and Hepatology, Department of Internal Medicine, Keio University School of Medicine, Tokyo, Japan; 3 Department of Pathology, Keio University School of Medicine, Tokyo, Japan

**Keywords:** pancreatic cancer, autoimmune pancreatitis, chronic pancreatitis, IgG4-related disease, inflammation

## Abstract

**INTRODUCTION:**

Although pancreatic cancer rarely co-occurs with autoimmune pancreatitis (AIP), distinguishing between AIP relapse and pancreatic cancer remains difficult, potentially leading to delayed diagnosis. A recent nationwide survey in Japan indicated that pancreatic cancer underlies a significant proportion of cancer-related deaths among patients with AIP.

**CASE PRESENTATION:**

Here, we present two cases of pancreatic cancer that initially mimicked AIP relapse. **Case 1:** An 89-year-old man with a long-standing history of pancreatic enlargement began steroid therapy for suspected AIP based on elevated serum IgG4 levels. Although IgG4 levels initially decreased following the treatment, they subsequently rose again, accompanied by worsening pancreatic swelling. Endoscopic ultrasound-fine-needle aspiration (EUS-FNA) revealed adenocarcinoma. **Case 2:** A 76-year-old woman with AIP, diagnosed based on focal pancreatic body enlargement and elevated IgG4, experienced multiple steroid-responsive relapses over 8 years. While tapering steroids, a new pancreatic nodule was detected on MRI, which was characterized by high signal intensity on diffusion-weighted imaging. Although the initial EUS-FNA was negative for carcinoma, a repeat biopsy 10 months later confirmed pancreatic cancer. Both patients underwent laparoscopic or robotic distal pancreatectomy with lymphadenectomy, and histopathological analysis confirmed pancreatic cancer arising in severely AIP-affected pancreatic tissue.

**CONCLUSIONS:**

In patients showing clinical or radiological worsening during AIP follow-up, repetitive diagnostic evaluations are warranted to facilitate the timely detection of underlying pancreatic cancer.

## Abbreviations

AIP
: autoimmune pancreatitis
CT
: computed tomography
EUS-FNA
: endoscopic ultrasound-fine-needle aspiration

## INTRODUCTION

Autoimmune pancreatitis (AIP) is a rare form of chronic pancreatitis characterized by elevated serum IgG4 levels, lymphoplasmacytic infiltration, and fibrosis, and is now considered a pancreatic manifestation of IgG4-related disease.^1–3)^ Type 1 AIP frequently presents with extrapancreatic lesions, such as sclerosing cholangitis, sclerosing sialadenitis, retroperitoneal fibrosis, chronic thyroiditis, and interstitial nephritis. Because AIP often appears as a pancreatic mass or causes obstructive jaundice, distinguishing it from pancreatic cancer at initial presentation can be challenging.^4)^ Moreover, pancreatic cancer that arises during the follow-up of AIP is even more difficult to differentiate, as its clinical and imaging findings can closely mimic AIP relapse,^5–7)^ potentially delaying diagnosis and treatment. Although such cases are rare, their timely detection is essential due to the poor prognosis of pancreatic cancer. In this report, we present two cases of pancreatic cancer that developed during long-term follow-up for AIP and were treated with radical resection, emphasizing the importance of repeated thorough examinations when imaging or clinical worsening is recognized.

## CASE PRESENTATION

### Case 1

**Fig. 1:**
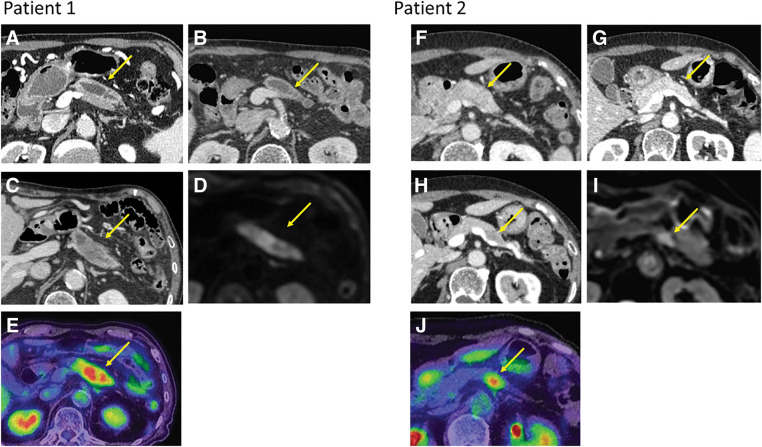
Changes in imaging results of the pancreas over time in two patients. (**A**, **F**) Contrast-enhanced CT scan images at AIP diagnosis revealing enlargement of the pancreatic body. (**B**, **G**) Contrast-enhanced CT scan images after steroid treatment showing improvement of pancreatic swelling. (**C**, **H**) Contrasted-enhanced CT scan images when pancreatic cancer was detected showing a hypodense mass in the pancreatic body. (**D**, **I**) Diffusion-weighted MRI revealing a high signal in the pancreatic body. (**E**, **J**) PET-CT revealed high uptake of fluorodeoxyglucose in the pancreatic body.

**Fig. 2:**
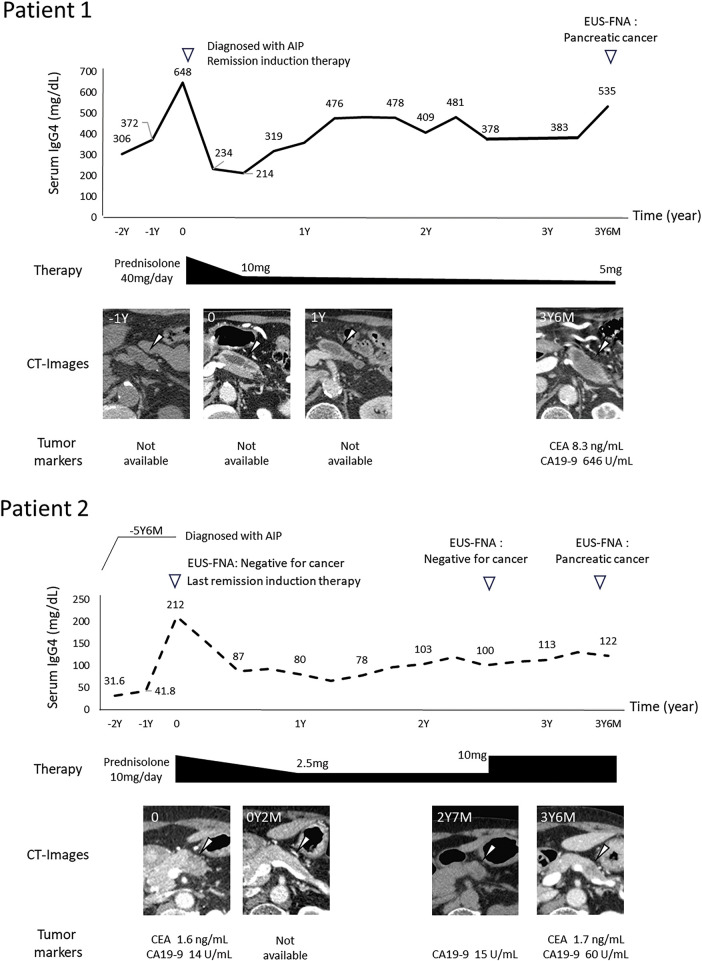
The overview of the clinical course in two cases.

An 89-year-old man had been under observation for decades after a computed tomography (CT) scan revealed an asymptomatic enlargement of the pancreas with a stone obstructing the pancreatic duct. During follow-up, the patient presented with swelling of the right submandibular gland and underwent submandibular adenectomy, which led to the diagnosis of IgG4-related chronic sclerosing sialadenitis. In the third postoperative year, the patient’s serum IgG4 level gradually increased, and the CT scan showed dilatation of the main pancreatic duct from the pancreatic body to the tail, leading to a diagnosis of AIP (**Fig. 1A**). The patient was administered 40 mg prednisolone as remission induction therapy after confirming the absence of cancer using endoscopic ultrasound-guided fine needle aspiration (EUS-FNA). Swelling of the pancreatic body improved (**Fig. 1B**) and the serum IgG4 levels rapidly decreased (**Fig. 2**), consistent with the typical course of AIP.

**Fig. 3:**
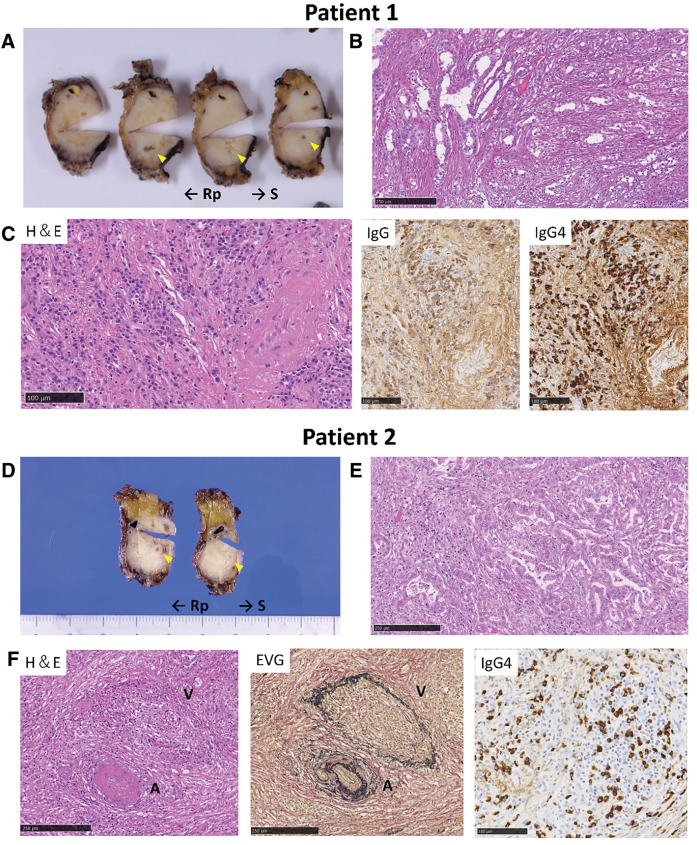
Pathological findings in the resected tissues of the pancreas of the two patients. (**A**, **D**) Macroscopic appearance of the resected distal pancreatic body; a whitish tumor was observed in the enlarged pancreatic body. Yellow arrowhead: main pancreatic duct. (**B**, **E**) H&E staining image of the pancreatic tissues; a transition zone from moderate to poor differentiation was observed in the invaded pancreatic parenchyma. Scale bar = 250 μm. (**C**, **F**) H&E and immunostaining of pancreatic tissues (H&E, Elastica van Gieson: EVG, IgG, and IgG4). A high degree of plasma cell and lymphocyte infiltration, storiform type fibrosis, obstructive phlebitis, and abundant IgG4-positive cells were observed. Scale bar = 100 μm. A, artery; H&E, Hematoxylin and Eosin; V, vein

During follow-up for AIP, although the patient presented with no specific symptom, an abdominal CT scan revealed worsening enlargement of the pancreatic body. Blood tests showed high serum IgG4 levels (383 mg/dL; normal range 11–121 mg/dL) and elevated levels of tumor markers, such as carcinoembryonic antigen (8.3 ng/mL; normal, <5 ng/mL), carbohydrate antigen 19-9 (646 U/mL; normal, 0–37 U/mL), Duke pancreatic monoclonal antigen (265 U/mL; normal, <150 U/mL), and s-pancreas-1 antigen (299 U/mL, normal, <30 U/mL). Contrast-enhanced CT of the chest, abdomen, and pelvis showed a 5-cm hypodense mass with a weak contrast effect in the pancreatic body and caliber irregularity in the splenic artery (**Fig. 1C**). T2-weighted MRI showed dilation of the main pancreatic duct running through the tumor, and diffusion-weighted abdominal MRI showed high signal intensity (**Fig. 1D**). Positron emission tomography (PET)-CT revealed high uptake of fluorodeoxyglucose (**Fig. 1E**), while EUS-FNA revealed adenocarcinoma cells. Subsequently, the patient underwent laparoscopic distal pancreatectomy with lymphadenectomy. The pancreas was overall hard, and the border between the normal pancreatic parenchyma and the tumor was difficult to recognize. Surgery was performed successfully, and the patient had an uneventful postoperative recovery. In the resected specimen, a whitish tumor was observed in the enlarged pancreatic body, and the tumor infiltrated almost the entire pancreatic body and tail (**Fig. 3A**). The surgical margins were negative for carcinoma. The walls of the main pancreatic duct and its surrounding areas were thickened with increased collagen fibers induced by AIP, and no stenosis of the main pancreatic duct was observed, despite the tumor being predominantly poorly differentiated. A transition zone from moderate-to-poor differentiation was observed (**Fig. 3B**), along with highly differentiated lesions on the caudal side of the pancreatic duct. In the pancreatic parenchyma, where the tumor was not involved, a high degree of plasma cell and lymphocyte infiltration, storiform fibrosis, obstructive phlebitis, and abundant IgG4-positive cells (≥100/HPF) was observed, consistent with AIP (**Fig. 3C**). The final diagnosis was invasive ductal carcinoma that was poorly differentiated and associated with type 1 autoimmune pancreatitis; pancreatic ductal adenocarcinoma (PDAC), Ptb, TS3 (28 × 20 × 52 mm), nodular, ductal adenocarcinoma, pT3, int, INFb, ly0, v2, ne3, mpd1 (10 mm), pCHX, pDUX, pS1(serosa), pRP1, pPV1(PVsp), pA1(Asp1), pN0 (0/18), pM0, pStageIIB (JPS 8th ed) and pT3, pN0, pM0, pStage IIA (UICC 8th ed). The patient received S-1 (tegafur, gimeracil, and oteracil potassium) as adjuvant chemotherapy; however, it was discontinued after one course owing to side effects, including tearing and diarrhea. Recurrence in the residual pancreas was observed at 10 months post operation, and palliative care was provided. The patient died 21 months after surgery.

### Case 2

A 76-year-old woman was treated with steroids for over 10 years following the diagnosis of Mikulicz’s disease, and was diagnosed with AIP 9 years ago owing to elevated serum IgG4 levels and the presence of a mass in the pancreatic body (**Figs. 1F** and **2**). During follow-up for AIP, the patient exhibited signs of suspected AIP exacerbation, such as an increase in the pancreatic body mass, which improved with steroid dose adjustments (**Fig. 1G**). While tapering the steroids off, a new 13-mm hypodense mass was detected in the pancreatic body (**Fig. 1H**), characterized by a high signal on DWI (**Fig. 1I**). The patient's serum CA19-9 level had been within the normal range and was 15 U/mL at the time. Although her serum IgG4 level was within the normal range of 119 mg/dL, it showed an increasing trend. EUS-FNA performed on the mass yielded negative results for malignancy. However, during careful follow-up, the mass gradually increased in size, and the serum CA19-9 level was elevated to 60 U/mL. Fluorodeoxyglucose (FDG) accumulation was observed using PET-CT (**Fig. 1J**). Therefore, a repeat EUS-FNA was performed 10 months later, which revealed adenocarcinoma. Subsequently, the patient underwent robotic distal pancreatectomy with lymphadenectomy. Intraoperatively, the pancreas was generally firm, with a well-defined hypoechoic mass in its body. While the surgery was successful, the patient developed an intraperitoneal abscess in the pancreatic remnant during the postoperative course, which resolved with percutaneous drainage. Histopathological examination of the resected specimen revealed a whitish tumor within the fibrotic pancreatic parenchyma affected by AIP (**Fig. 3D**). Tumor invasion was observed near the splenic vein and artery; however, no vascular invasion was seen. A transition from moderately differentiated to poorly differentiated carcinoma was noticed (**Fig. 3E**). The background pancreatic parenchyma showed extensive plasma cell and lymphocyte infiltration, storiform fibrosis, obstructive phlebitis, and abundant IgG4-positive cells (≥10/HPF), consistent with AIP (**Fig. 3F**). The final diagnosis was moderately differentiated invasive ductal carcinoma associated with type 1 AIP; PDAC, Pb, TS2 (10 × 20 × 20 mm), nodular type, pT1c, int, INFb, ly0, v1a, ne0, mpd1 (5 mm), pCHX, pDUX, pS0, pRP0, pPV0, pA0, pN0 (0/35), pM0, pStage IA (JPS 8th ed.), and pT1, pN0, pM0, and pStage IA (UICC 8th ed.). The patient received adjuvant chemotherapy with S-1 for 3 months postoperatively; however, treatment was discontinued due to severe anorexia. The patient remains recurrence-free 1 year after surgery.

## DISCUSSION

AIP is a relatively new disease and its long-term prognosis, including its relationship with pancreatic cancer, remains unclear.^8)^ A nationwide survey was conducted every 4 years in Japan, including 1474 patients with AIP surveyed in 2016.^3)^ According to the report, malignancies were observed in 242 patients during the observation period, with high frequency of gastric, colorectal, bladder, and pancreatic cancers in 18 patients (1.2%). Of the 242 malignancies, 17 resulted in cancer-related deaths, 8 of which (47%) were owing to pancreatic cancer. Moreover, a multicenter retrospective cohort study in Japan involving 1364 patients with type 1 AIP reported 19 cases of pancreatic cancer over a median follow-up of 5.6 years, representing an incidence rate 3.22-fold higher than that in the general population. The study identified pancreatic cancer as a critical determinant of survival.^9)^ Considering these facts, the occurrence of pancreatic cancer during clinical observation of AIP should always be a concern, despite it being relatively infrequent.

A link between carcinogenesis and chronic inflammation has been documented in various cancers, with chronic pancreatitis being recognized as a significant carcinogenic factor.^10)^ Recent studies have shown that some patients with AIP exhibit a disease course resembling that of chronic pancreatitis, including the formation of pancreatic stones and fibrosis over time, as seen in the current case.^11)^ Therefore, chronic inflammation associated with AIP could possibly promote carcinogenesis, similar to that in ordinary chronic pancreatitis. Gupta et al. reported a high frequency of pancreatic intraepithelial neoplasia (PanIN) lesions, which are considered precancerous, in the pancreatic tissues of patients with type 1 AIP, at rates comparable to those in patients with chronic pancreatitis (64% vs. 63%).^7)^ Additionally, Kamisawa et al. identified mutations in KRAS, a potent carcinogenic factor, in the pancreatic tissues of patients with AIP,^12)^ whereas Kinugawa et al. detected methylation of tumor suppressor genes in these tissues.^13)^ Although further research would be required to elucidate the relationship between AIP and pancreatic cancer, the findings suggested that AIP has carcinogenic potential. A previously reported model of pancreatic cancer development and progression estimated that an average of 11.7 years from the onset of carcinogenesis is required for *in situ* pancreatic cancer to develop, suggesting that pancreatic cancer may develop during long-term follow-up of AIP.^14,15)^ In Case 1, histopathological examination revealed well-differentiated lesions in the main pancreatic duct, which transitioned to poorly differentiated areas, indicating stepwise progression of pancreatic cancer. Previous reports of pancreatic cancer diagnosed during AIP follow-up are summarized in **Table 1**.

**Table 1:** Clinical features of patients with pancreatic cancer that developed during the course of AIP

Auto-immune pancreatitis									Pancreatic cancer				
No	Author year [ref.]	Age (year)	Sex	Serum IgG4 (mg/dL)	Swelling region	Stone	Pathological findings (IgG4 count/HPF)	Steroid therapy	Interval from the diagnosis of AIP	Region	Imaging findings	EUS-FNA	Treatment
1	Ghazale 2007^5)^	72	M	N/A	Ph	N/A	LI, SF, OP (>30 /HPF)	−	5 y	Pb	Peritoneal-metastasis	+	N/A
2	Fukui 2008^6)^	80	M	154	Ph	N/A	N/A	+	3 y	Pb	Pancreatic mass MPD stenosis Peritoneal-metastasis	+	Chemotherapy
3	Gupta 2012^7)^	73	M	N/A	N/A	N/A	SF, OP (>50 /HPF)	N/A	10 y	Pt	Pancreatic mass	+	N/A
4	Gupta 2012^7)^	69	M	147	Ph	N/A	LI, SF (16 /HPF)	+	8 y	Ph	Pancreatic mass MPD dilation	+	N/A
5	Ikeura 2014^18)^	61	F	338	Ph	N/A	LI, SF (N/A)	+	2 y 7 m	Ph	Pancreatic mass	+	PD
6	Ikeura 2014^18)^	39	F	N/A	Pt	N/A	LI (N/A)	+	15 y 6 m	Pb	Pancreatic mass	+	DP
7	Ikeura 2014^18)^	80	M	154	Ph	N/A	N/A	+	5 y 7 m	Pb	Pancreatic mass MPD dilation Peritoneal- metastasis	+	Chemotherapy
8	Ishikawa 2020^19)^	72	M	N/A	Ph, Pb, Pt	N/A	LI (N/A)	+	1 y 5 m	Pb	Pancreatic mass MPD dilation	N/A	N/A
9	Ishikawa 2020^19)^	72	M	N/A	Ph, Pb, Pt	+	LI (N/A)	+	1y 10 m	Pt	Pancreatic mass MPD dilation	N/A	N/A
10	Ichikawa 2023^22)^	70	M	281	Pb, Pt	−	LI, SF (>10 /HPF)	−	3 y 7 m	Pb	Pancreatic mass MPD dilation Liver metastasis	+	Chemotherapy
11	Ichikawa 2023^22)^	70	M	297	Ph	−	LI, SF (>10 /HPF)	+	1 y 6 m	Ph	Pancreatic mass IVC invasion	+	Chemotherapy
Case 1		89	M	383	Pb, Pt	+	LI, SF, OP (>100 /HPF)	+	3 y 6 m	Pb, Pt	Pancreatic mass	+	DP
Case 2		76	F	212	Pb, Pt	−	LI, SF, OP (>10 /HPF)	+	9 y	Pb	Pancreatic mass	+	DP

DP, distal pancreatectomy; EUS-FNA, endoscopic ultrasound-guided fine needle aspiration; HPF, high-power field; IVC, inferior vena cava; LI, lymphocyte infiltration; MPD, main pancreatic duct; N/A, not available; OP, obliterative phlebitis; Pb, pancreatic body; PD, pancreaticoduodenectomy; Ph, pancreatic head; Pt, pancreatic tail; SF, storiform type fibrosis.

In **Table 1**, the interval between the diagnosis of AIP and the diagnosis of pancreatic cancer varied widely, ranging from less than 2 years to over 15 years. Similarly, a Japanese multicenter retrospective cohort study reported that pancreatic cancer frequently occurred within 2 years or after 5 years of an AIP diagnosis.^9)^ This variability may reflect differences in the progression rate of multistep pancreatic carcinogenesis, influenced by the severity of inflammation and the treatment administered for AIP. Furthermore, repeated imaging studies are often performed in the early phase following AIP onset to evaluate treatment response and to screen for other manifestations of IgG4-related disease, which may contribute to the early detection of pancreatic cancer. These repetitive screening tests contribute to the detection of cancers other than pancreatic cancer, and subsequently influence the emergence of the hypothesis that AIP is a tumor-associated syndrome.^16,17)^

Most cases of pancreatic cancer that develop with AIP are treated with steroids.^6,18,19)^ While some reports have raised concerns that the immunosuppressive effects of steroids might promote carcinogenesis,^6)^ patients receiving steroid maintenance therapy have been shown to have a reduced risk of cancer and improved survival than those who do not.^9)^ AIP has a high recurrence rate of 20%–40%, even in patients receiving steroid therapy, with 20%–30% of recurrences occurring during maintenance therapy.^20)^ Consequently, worsening pancreatic conditions are often interpreted as a relapse of AIP, leading to intensified steroid treatment. This is particularly common in patients with a prior AIP diagnosis who are receiving long-term care. Furthermore, pancreatic cancer associated with AIP may show partial improvements in imaging results with steroid therapy, making the diagnosis of pancreatic cancer even more challenging.^21)^ Previous reports, listed in **Table 1**, documented the patients with peritoneal metastasis or liver metastasis detected at the time of pancreatic cancer diagnosis,^5,6,18,22)^ rendering them inoperable. As demonstrated in the clinical course of Patients 1 and 2, as well as in the case reported by Ishikawa et al.,^19)^ elevated serum IgG4 levels during follow-up cannot be definitively interpreted as a relapse of AIP. Similarly, serum CA19-9 levels may also show mild to moderate elevation during AIP relapse, and thus cannot serve as a definitive diagnostic tool. However, in cases where serum CA19-9 levels rise despite steroid therapy during the course of AIP treatment (e.g., Patient 2; Case 2 in **Table 1**), the possibility of concomitant pancreatic cancer should be carefully considered. The main features in cases that developed cancer during AIP follow-up were the formation of pancreatic tumors and dilation of the main pancreatic duct; however, such radiographic findings are often perceived as AIP.^23)^ Various imaging modalities, including US, CT, MRI, and PET-CT, have been utilized to differentiate AIP from pancreatic cancer. However, no definitive diagnostic tool has been established yet. The difficulty in differentiation arises from the prevalence of AIP in older adult patients with pre-existing pancreatic atrophy, along with the variability in the extent of inflammation and fibrosis, which can sometimes mimic the imaging features of pancreatic cancer. Characteristic findings of AIP, such as diffuse pancreatic enlargement, capsule-like rim, delayed enhancement, multiple pancreatic masses, and the duct-penetrating sign, are useful for distinguishing initial pancreatic masses.^24,25)^ However, these features are unreliable in identifying pancreatic cancer that develops during the course of AIP. In Patient 1, diffuse pancreatic enlargement and a capsule-like rim, typical of AIP, were still evident at the time of pancreatic cancer diagnosis. Although the duct-penetrating sign strongly suggests AIP over pancreatic cancer,^26)^ the patient’s main pancreatic duct diameter was preserved owing to severe fibrosis of the pancreatic parenchyma caused by AIP. Thus, in patients with a long history of AIP, the duct-penetrating sign may not be a definitive indicator. In addition to qualitative CT and MRI findings, quantitative evaluations using diffusion-weighted MRI and PET-CT were explored. A systematic review of the quantitative utility of ADC values in diffusion-weighted MRI reported statistically significant differences in ADC values between AIP and pancreatic cancer in eight studies. However, five studies found significantly lower ADC values in AIP while three reported lower values in pancreatic cancer.^27)^ Similarly, reports on FDG uptake values in PET-CT showed inconsistent results. While some studies reported that the standardized uptake max value (SUVmax) in the early and delayed phases was significantly higher in pancreatic cancer than in AIP,^28)^ Ozaki et al. reported no such difference.^29)^ Additionally, Lee et al. compared FDG uptake in pancreatic lesions between patients with AIP and those with pancreatic cancer and found no significant difference in overall frequency.^30)^

Differentiating between a relapse of AIP and pancreatic cancer based solely on laboratory findings or imaging studies remains challenging. Therefore, histopathological examination is currently recommended when diagnostic uncertainty persists. Previous reports on the utility of EUS-FNA and fine-needle biopsy (FNB) for the differential diagnosis of AIP and pancreatic cancer have demonstrated a diagnostic yield of 55.8% for FNA and 87.2% for FNB in cases of AIP.^31)^ By contrast, the sensitivity of FNA for detecting pancreatic cancer was reported to be as high as 92.2%.^32)^ Furthermore, in patients with suspected pancreatic cancer who initially received a negative result from EUS-FNA, serial EUS-FNA procedures was shown to improve diagnostic accuracy.^33)^ Thus, histopathological evaluation by EUS-FNA appears to be valuable in excluding malignancy in such clinical contexts.

Currently, no established consensus defines the optimal monitoring strategy for pancreatic cancer during follow-up in patients with AIP. However, considering that the incidence of pancreatic cancer in AIP patients is less than five times higher than in the general population, a surveillance approach similar to that for familial pancreatic cancer may be appropriate, where the relative risk increases by approximately 4.5-fold in individuals.^34)^ Specifically, 6- to 12-month imaging intervals using MRI or EUS are reasonable when no suspicious findings are present. Closer follow-up within 3 months and histopathological assessment are advisable if any notable changes are detected, such as progressive pancreatic enlargement, the emergence of new nodular lesions, or elevated serum CA19-9 levels during steroid therapy.^35)^ Repeated biopsy evaluations are crucial to confirm or exclude carcinoma and are particularly important for lesions in the pancreatic head, where the risk of needle tract seeding is minimal.^36)^ The current study underscored the importance of diligent follow-up for AIP and the need for thorough malignancy evaluation when imaging findings deteriorate, even in the context of a prior AIP diagnosis.

## CONCLUSIONS

AIP, over a relatively long period, can be complicated with pancreatic cancer. Even if diagnosed in the past, a repetitive investigation for possible pancreatic cancer should be conducted when AIP worsens.

## ACKNOWLEDGMENTS

We would like to thank Editage for English language editing.

## DECLARATIONS

### Funding

No funding was received for this case report.

### Authors’ contributions

HK interpreted the patient data based on the case notes and drafted the manuscript.

MK performed the surgery and supervised the manuscript.

EI treated the autoimmune pancreatitis and performed EUS-FNA.

YM and AU evaluated the pathological findings.

All other members contributed equally to the medical treatment.

All authors have read and approved the manuscript.

### Availability of data and materials

The data of the current study are available from the corresponding author upon reasonable request.

### Ethics approval and consent to participate

This study was approved by the ethics committee of the Keio University School of Medicine (Approval No.: 20120443).

### Consent for publication

Written informed consent was obtained from the patients for the publication of this report and accompanying images.

### Competing interests

The authors declare no competing interests.

## References

[1] Okazaki K Chiba T. Autoimmune related pancreatitis. Gut 2002; 51: 1–4. 12077078 10.1136/gut.51.1.1 PMC1773283

[2] Shimosegawa T Chari ST Frulloni L International consensus diagnostic criteria for autoimmune pancreatitis: guidelines of the International Association of Pancreatology. Pancreas 2011; 40: 352–8. 21412117 10.1097/MPA.0b013e3182142fd2

[3] Masamune A Kikuta K Hamada S Nationwide epidemiological survey of autoimmune pancreatitis in Japan in 2016. J Gastroenterol 2020; 55: 462–70. 31872350 10.1007/s00535-019-01658-7

[4] Wolske KM Ponnatapura J Kolokythas O Chronic pancreatitis or pancreatic tumor? A problem-solving approach. Radiographics 2019; 39: 1965–82. 31584860 10.1148/rg.2019190011

[5] Ghazale A Chari S. Is autoimmune pancreatitis a risk factor for pancreatic cancer? Pancreas 2007; 35: 376. 10.1097/MPA.0b013e318073ccb8 18090248

[6] Fukui T Mitsuyama T Takaoka M Pancreatic cancer associated with autoimmune pancreatitis in remission. Intern Med 2008; 47: 151–5. 18239323 10.2169/internalmedicine.47.0334

[7] Gupta R Khosroshahi A Shinagare S Does autoimmune pancreatitis increase the risk of pancreatic carcinoma? A retrospective analysis of pancreatic resections. Pancreas 2013; 42: 506–10. 23271394 10.1097/MPA.0b013e31826bef91

[8] Wallace ZS Wallace CJ Lu N Association of IgG4-related disease with history of malignancy. Arthritis Rheumatol 2016; 68: 2283–9. 27273903 10.1002/art.39773 PMC7524567

[9] Takikawa T Kikuta K Sano T Maintenance steroid therapy is associated with decreased risk of malignancy and better prognosis of patients with autoimmune pancreatitis: A multicenter cohort study in Japan. Pancreatology 2024; 24: 335–42. 38336506 10.1016/j.pan.2024.01.008

[10] Okada F. Inflammation-related carcinogenesis: current findings in epidemiological trends, causes and mechanisms. Yonago Acta Med 2014; 57: 65–72. 25324587 PMC4198572

[11] Maruyama M Watanabe T Kanai K Autoimmune pancreatitis can develop into chronic pancreatitis. Orphanet J Rare Dis 2014; 9: 77. 24884922 10.1186/1750-1172-9-77 PMC4038704

[12] Kamisawa T Tsuruta K Okamoto A Frequent and significant K-ras mutation in the pancreas, the bile duct, and the gallbladder in autoimmune pancreatitis. Pancreas 2009; 38: 890–5. 19752775 10.1097/MPA.0b013e3181b65a1c

[13] Kinugawa Y Uehara T Sano K Methylation of tumor suppressor genes in autoimmune pancreatitis. Pancreas 2017; 46: 614–8. 28196014 10.1097/MPA.0000000000000804

[14] Yachida S Jones S Bozic I Distant metastasis occurs late during the genetic evolution of pancreatic cancer. Nature 2010; 467: 1114–7. 20981102 10.1038/nature09515 PMC3148940

[15] Hanada K Amano H Abe T. Early diagnosis of pancreatic cancer: current trends and concerns. Ann Gastroenterol Surg 2017; 1: 44–51. 29863166 10.1002/ags3.12004 PMC5881352

[16] Shiokawa M Kodama Y Yoshimura K Risk of cancer in patients with autoimmune pancreatitis. Am J Gastroenterol 2013; 108: 610–7. 23318486 10.1038/ajg.2012.465

[17] Okamoto A Watanabe T Kamata K Recent updates on the relationship between cancer and autoimmune pancreatitis. Intern Med 2019; 58: 1533–9. 30713326 10.2169/internalmedicine.2210-18 PMC6599917

[18] Ikeura T Miyoshi H Uchida K Relationship between autoimmune pancreatitis and pancreatic cancer: a single-center experience. Pancreatology 2014; 14: 373–9. 25278307 10.1016/j.pan.2014.04.029

[19] Ishikawa T Kawashima H Ohno E Risks and characteristics of pancreatic cancer and pancreatic relapse in autoimmune pancreatitis patients. J Gastroenterol Hepatol 2020; 35: 2281–8. 32583452 10.1111/jgh.15163

[20] Kamisawa T Okazaki K Kawa S Amendment of the Japanese Consensus Guidelines for Autoimmune Pancreatitis, 2013 III. Treatment and prognosis of autoimmune pancreatitis. J Gastroenterol 2014; 49: 961–70. 24639058 10.1007/s00535-014-0945-z

[21] Sakashita F Tanahashi T Yamaguchi K Case of pancreatic tail cancer associated with autoimmune pancreatitis. Jpn J Gastroenterol Surg 2006; 39: 78–83. (in Japanese)

[22] Ichikawa H Iwashita T Senju A Development of pancreatic cancer during the follow-up of autoimmune pancreatitis: a report of two cases. Intern Med 2024; 63: 949–56. 37612085 10.2169/internalmedicine.2086-23 PMC11045376

[23] Hsu WL Chang SM Wu PY Localized autoimmune pancreatitis mimicking pancreatic cancer: Case report and literature review. J Int Med Res 2018; 46: 1657–65. 29332510 10.1177/0300060517742303 PMC6091832

[24] Yoon SB Jeon TY Moon S-H Differentiation of autoimmune pancreatitis from pancreatic adenocarcinoma using CT characteristics: a systematic review and meta-analysis. Eur Radiol 2023; 33: 9010–21. 37466708 10.1007/s00330-023-09959-5

[25] Yoon SB Jeon TY Moon S-H Systematic review and meta-analysis of MRI features for differentiating autoimmune pancreatitis from pancreatic adenocarcinoma. Eur Radiol 2022; 32: 6691–701. 35486167 10.1007/s00330-022-08816-1

[26] Ichikawa T Sou H Araki T Duct-penetrating sign at MRCP: usefulness for differentiating inflammatory pancreatic mass from pancreatic carcinomas. Radiology 2001; 221: 107–16. 11568327 10.1148/radiol.2211001157

[27] Ha J Choi SH Kim KW MRI features for differentiation of autoimmune pancreatitis from pancreatic ductal adenocarcinoma: A systematic review and meta-analysis. Dig Liver Dis 2022; 54: 849–56. 34903501 10.1016/j.dld.2021.11.013

[28] Zhang J Jia G Zuo C ^18^F- FDG PET/CT helps differentiate autoimmune pancreatitis from pancreatic cancer. BMC Cancer 2017; 17: 695. 29061130 10.1186/s12885-017-3665-y PMC5654006

[29] Ozaki Y Oguchi K Hamano H Differentiation of autoimmune pancreatitis from suspected pancreatic cancer by fluorine-18 fluorodeoxyglucose positron emission tomography. J Gastroenterol 2008; 43: 144–51. 18306988 10.1007/s00535-007-2132-y

[30] Lee TY Kim MH Park DH Utility of ^18^F-FDG PET/CT for differentiation of autoimmune pancreatitis with atypical pancreatic imaging findings from pancreatic cancer. AJR Am J Roentgenol 2009; 193: 343–8. 19620430 10.2214/AJR.08.2297

[31] Yoon SB Moon SH Song TJ Endoscopic ultrasound-guided fine needle aspiration versus biopsy for diagnosis of autoimmune pancreatitis: Systematic review and comparative meta-analysis. Dig Endosc 2021; 33: 1024–33. 33030283 10.1111/den.13866

[32] de Pretis N Crinò SF Frulloni L. The role of EUS-guided FNA and FNB in autoimmune pancreatitis. Diagnostics (Basel) 2021; 11: 1653. 34573995 10.3390/diagnostics11091653 PMC8470670

[33] Lisotti A Frazzoni L Fuccio L Repeat EUS-FNA of pancreatic masses after nondiagnostic or inconclusive results: systematic review and meta-analysis. Gastrointest Endosc 2020; 91: 1234–41.e4. 32006546 10.1016/j.gie.2020.01.034

[34] Klein AP Brune KA Petersen GM Prospective risk of pancreatic cancer in familial pancreatic cancer kindreds. Cancer Res 2004; 64: 2634–8. 15059921 10.1158/0008-5472.can-03-3823

[35] Canto MI Harinck F Hruban RH International Cancer of the Pancreas Screening (CAPS) Consortium summit on the management of patients with increased risk for familial pancreatic cancer. Gut 2013; 62: 339–47. 23135763 10.1136/gutjnl-2012-303108 PMC3585492

[36] Kojima H Kitago M Iwasaki E Peritoneal dissemination of pancreatic cancer caused by endoscopic ultrasound-guided fine needle aspiration: a case report and literature review. World J Gastroenterol 2021; 27: 294–304. 33519143 10.3748/wjg.v27.i3.294 PMC7814364

